# Biochemical and histopathological study in rats intoxicated with carbontetrachloride and treated with camel milk

**DOI:** 10.1186/2193-1801-2-57

**Published:** 2013-02-18

**Authors:** Thnaian Althnaian, Ibrahim Albokhadaim, Sabry M El-Bahr

**Affiliations:** 1 Department of Anatomy, College of Veterinary Medicine and Animal Resources, King Faisal University, Al-Ahsa, Saudi Arabia; 2 Department of Physiology, Biochemistry and Pharmacology (Physiology), College of Veterinary Medicine and Animal Resources, King Faisal University, Al-Ahsa, Saudi Arabia; 3 Department of Physiology, Biochemistry and Pharmacology (Biochemistry), College of Veterinary Medicine and Animal Resources, King Faisal University, Al-Ahsa, Saudi Arabia; 4 Department of Biochemistry, Faculty of Veterinary Medicine, Alexandria University, Alexandria, Egypt

**Keywords:** Camel milk, Blood, Liver, Biochemistry, Histopathology

## Abstract

The unique characters of camel’s milk make it used extensively in the field of medicine as anti-microbial, anti-diabetic and hepatoprotective agent. The lack of studies demonstrating the protective effect of camel’s milk against hepatotoxic compound was the main reason beyond the conduction of the current experiment which aimed to investigate the protective effects of camel’s milk against carbontetrachloride (CCl_4_) induced hepatotoxicity. Therefore, 24 rats were fed on standard diet and divided into four groups. Rats of the first group and second groups were injected i/p with paraffin oil and received either tap water (control 1) or camel’s milk (control 2), respectively. Rats of the third and fourth groups were injected i/p with CCl_4_ and received either tap water or camel’s milk, respectively. At the end of the experiment (5 weeks), blood and liver samples were collected for biochemical and histopathological analysis. The present findings revealed that, CCl_4_ elevated serum enzyme activities of liver and some biochemical parameters, but these effects were prevented by the treatment of rats with camel milk. Histopathologically, a great amount of mononuclear cells infiltration, necrotic cells and few fibroblasts were observed in liver of CCl_4_ treated group. The present study concluded that camel milk treatment may play a protective role against CCl_4_-induced liver damages in rats. These protective effects were in the form of improving of liver enzyme activities, blood biochemical parameters and histological picture of liver of intoxicated rats. In the future, examination of the liver protective effect of camel milk against CCl_4_ in dose dependant manner could be investigated.

## Introduction

The liver is responsible for metabolism and detoxification of the most of components that enter the body (Nunez 2006). Carbon tetrachloride (CCl_4_) is a highly toxic chemical agent, the most famous drug used to induce liver damage experimentally. Histopathological sectioning of the liver tissues indicated that, CCl_4_ induced fibrosis, cirrhosis and hepatocarcinoma (Junnila et al. 2000; Karakus et al. 2011). The toxic effect of CCl_4_ is attributed to trichloromethyl radical produced during oxidative stress (Stoyanovsky and Cederbaum 1999). The number of infiltrated neutrophils, macrophages, Kupffer cells, lymphocytes and natural killer cells are significantly increased after liver injury induced by hepatotoxins such as CCl_4_. It induced activation of liver resident macrophages and/or chemoattraction of extrahepatic cells (e.g. neutrophils and lymphocytes; Ramadori et al. 2004). The activated macrophages are released and contributed to liver fibrosis, inflammation and injury (Canbay et al. 2004; 2007). Once the liver became injured, its efficient treatment with famous chemical drugs is limited (Lee et al. 2007). Therefore, interest concerned the use of alternative medicines for the treatment of hepatic disease has been arisen. The presence of peptides and proteins in camel’s milk exhibits its biological activities that have beneficial effect on many bioprocesses as digestion, absorption, growth and immunity (Yagil et al. 1984; Korhonen and Pihlanto 2001). Furthermore, camel’s milk can be stored at room temperature longer period than milk from other animals (Omer and Eltinay 2009). The most described uses of camel’s milk are as drug against autoimmune diseases, dropsy, jaundice, spleenomegaly, tuberculosis, asthma, anemia, piles and diabetes (Rao et al. 1970). Antimicrobial activities of camel’s milk proteins were also investigated (El-Agamy et al. 1992). In addition, camel’s milk has antitoxic effect against cadmium chloride (Al-Hashem et al. 2009; Dallak 2009), CCl_4_ (Khan and Alzohairy 2011), Cisplatin (Afifi 2010), Paracetamol (Al-Fartosi et al. 2011), Aluminum chloride (Al-Hashem 2009). Although, Khan and Alzohairy (2011) studied the protective effect of camel’s milk against CCl_4_ induced hepatotoxicity, biochemical parameters such as Kidney biomarkers and lipoprotein profiles were not fully investigated. Therefore, in the present study, we investigated the protective effects of camel’s milk against CCl_4_-induced hepatotoxicity in rats by assaying liver and kidney functions, lipid profiles and histopathology of liver tissues.

## Materials and methods

### Chemicals and kits

Diagnostic kits for serum total proteins, albumin, total lipid, triglyceride, total cholesterol, HDL-c, LDL-c, VLDL-c, alanine aminotransferase (ALT) and aspartate amino transferase (AST), alkaline phosphatase (ALP), blood urea nitrogen (BUN), uric acid and creatinine were purchased from ELIPSE, United diagnostic industry, UDI, Dammam, Saudi Arabia. Paraffin oil, carbon tetrachloride (Spectrosol® BHD chemicals ltd pool, England) and other chemicals and solvents were of highest grade commercially available.

### Camel’s milk

Camel’s milk samples were collected daily early in the morning from camel farm in Camel research center, King Faisal University, Al-Ahsa (Estern Providence), Saudi Arabia. Milk was collected from camels by hand milking. The samples were collected in sterile screw bottles and kept in cool boxes until transported to the laboratory. The rats were given this fresh milk (100 mL/24 h/cage) as such without any further treatment.

### Animals and treatment

A total of 24 albino rats (200–250 g) were obtained from Laboratory house of college of Veterinary Medicine and Animal Resources, King Faisal University, Al-Ahsa, Saudi Arabia and acclimated for 10 days before starting the experiment. All animals were housed in standard cages (6 rats/cage), feeding with standard laboratory diet and tap water *ad libitum*. The experimental animals were housed in air-conditioned rooms at 21-23°C and 60-65% of relative humidity and kept on a 12 h light/12 h dark cycle. The animals received humane care in accordance with the Guide for the Care and Use of Laboratory Animals, published by ethics of scientific research committee of King Faisal University, Saudi Arabia.

### Induction of hepatotoxicity by CCl_4_

Liver toxicity was induced by the intraperitoneal injection of CCl_4_ (1 ml/kg b.wt.), 1:1 diluted with paraffin oil, for two successive days of the experiment (Khan and Alzohairy 2011).

### Experimental groups and protocol

The rats were divided randomly into 4 groups comprising 6 rats in each group and fed the same diet throughout the experimental period. The experimental design is described as fellow:

Group I: Rats fed only with basal diet and tap water and injected i/p with Paraffin oil, this group served as control 1.

Group II: Rats fed normal basal diet, injected i/p with Paraffin oil and received camel’s milk (100 ml/24 h/cage) as their sole source of drinking water, this group served as control 2.

Group III: Rats fed basal diet and tape water and intoxicated with CCl_4_ (1 ml/kg b.wt.), 1:1 diluted with paraffin oil on first two days of the experiment.

Group IV: Rats fed basal diet and intoxicated with CCl_4_ (1 ml/kg b.wt.), 1:1 diluted with paraffin oil on first two days of the experiment and then treated with camel’s milk (100 ml/24 h/cage) as their sole source of drinking water.

### Blood and tissue collection

At the end of the experiment, the overnight fasted animals (the control and experimental animals) were sacrificed under light ether anesthesia. Blood samples were collected by cardiac puncture before incision of the abdomen; 5 ml of blood samples were collected in plain tubes, serum was collected and frozen at —30°C until the time of analysis. Liver tissues were cut in small pieces and immersed in neutral buffered formalin 10% for histopathology.

### Biochemical analysis

Commercial diagnostic kits (United Diagnostic Industry, UDI, Dammam, Saudi Arabia) were used for determination of Glucose (EP37L-660), total proteins (EP56-660), Albumin (EP03-570), ALT (EP07-500), AST (EP15-500), ALP (EP04L-660), ACP (EP02-295), BUN (EP20-420), Uric acid (EP61-620), Creatinine (EP33K-660), CK (EP28-310), TAG (EP59-660), cholesterol (EP24-660), Calcium (EP22-660), Phosphorus (EP46-660), Magnesium (EP50-660), Chloride (EP27-500) on ELIPSE full automated chemistry analyzer (Rome, Italy). Concentration of the biochemical constituents was calculated according to the manufacture instruction.

### Histopathological examinations

Liver tissues were cut in small pieces and immersed in neutral buffered formalin for 24 h. The fixed tissues were processed routinely, embedded in paraffin, sectioned, deparaffinized and rehydrated using the standard techniques (Bancroft and Gamble 2002). The extent of CCl_4_-induced necrosis was evaluated by assessing the morphological changes in the liver sections stained with hematoxylin and eosin (H and E), using standard techniques.

### Statistical analysis

Results were expressed as Means ± standard error of mean (SEM). The significance of differences was calculated by using student t-test, p < 0.05 was considered statistically significant.

## Results

### Biochemical findings

The activities of AST, ALT and ALP were estimated in serum samples as the liver function biomarkers. These results are given in Table 1. The CCl_4_ treatment markedly affected the liver specific enzymes. It was found that a significant (p < 0.05) increase in serum AST, ALT and ALP activities of CCl_4_ treated rats. This result suggests that these hepatic biomarkers were elevated in the serum due to release of the enzymes from damaged liver. However a significant decrease (p < 0.05) was observed in the respective serum activities of rats given Camel milk + CCl_4_ compared with CCl_4_ treated group. In the other hand, the activities of ACP showed insignificant changes (p > 0.05) in all treated groups. The levels of serum kidney biomarkers of rats in all groups are presented in Table 2. BUN level were decreased significantly (p < 0.05) in the CCl_4_ treated group compared with the control one. Uric acid levels were increased significantly (p < 0.05) in the CCl_4_ treated group compared with the control. However, creatinine level remained unchanged (p > 0.05) in all treated groups. The profiles of serum proteins, lipids and glucose are presented in Table 3. A significant decrease (p < 0.05) in serum glucose, total proteins and albumin of CCl_4_ treated rats (p < 0.05) was observed. However, the values of glucose and total proteins were corrected towards the control values in rats intoxicated with CCl_4_ and treated with camel milk. Cholesterol and triglyceride levels were increased significantly (p < 0.05) in the CCl_4_ group compared with the control. There were significant (p < 0.05) decreases in cholesterol and triglyceride levels in the camel milk + CCl_4_ treated rats compared with the CCl_4_ treated rats. A significant change was not observed (p > 0.05) in calcium, phosphorus, magnesium and chloride levels of all treated groups (Table 4).

**Table 1:** **Effects of CCl**_**4**_**and camel milk on serum enzyme activities of liver in rat**

Groups	Parameters			
	AST IU/L	ALT IU/L	ALP IU/L	ACP IU/L
C	118.9 ± 06.0	29.2 ± 0.1	161.2 ± 05.7	7.5 ± 0.2
CM	111.6 ± 05.0	32.2 ± 1.7	214.8 ± 00.8	7.5 ± 0.6
CCl4	130.8 ± 03.7^a^	47.4 ± 4.0^a^	372.7 ± 10.0^a^	7.0 ± 0.1
CM + CCl4	125.6 ± 14.0^b^	34.7 ± 3.5^b^	272.0 ± 09.0^b^	7.2 ± 0.7

Each value represents the mean ± standard error of means (SEM) of 6 rats.

^a^ Significantly different from control group(*p* < 0.05).

^b^ Significantly different from CCl_4_ group(*p* < 0.05).

*C*: Control, *CM*: camel milk, *CCl*_*4*_: Carbon tetrachloride.

**Table 2:** **Effects of CCl**_**4**_**and camel milk on kidney markers in rat**

Groups	Parameters		
	BUN mg/dl	Uric acid mg/dl	Creatinine mg/dl
C	11.1 ± 0.2	1.8 ± 0.20	0.4 ± 0.05
CM	12.1 ± 0.1	1.4 ± 0.01	0.4 ± 0.05
CCl4	9.2 ± 0.1^a^	2.6 ± 0.07^a^	0.4 ± 0.10
CM + CCl4	8.7 ± 0.3^a^	2.6 ± 0.20^a^	0.4 ± 0.20

Each value represents the mean ± standard error of means (SEM) of 6 rats.

^a^ Significantly different from control group(*p* < 0.05).

*C*: Control, *CM*: camel milk, *CCl*_*4*_: Carbon tetrachloride.

**Table 3:** **Effects of CCl**_**4**_**and camel milk on protein, lipid and glucose profiles in rat**

Groups	Parameters				
	Glucose mg/dl	Total protein g/dl	Albumin g/dl	Triacylglycerol mg/dl	Cholesterol mg/dl
C	317 ± 1.0	5.6 ± 0.1	3.4 ± 0.20	54.7 ± 5.0	44.7 ± 4.0
CM	210 ± 7.1	5.3 ± 0.4	2.9 ± 0.04	47.7 ± 3.0	46,3 ± 2.0
CCl4	227 ± 2.1^a^	4.8 ± 0.1^a^	2.4 ± 0.10^a^	72.4 ± 6.0^a^	50.3 ± 0.7^a^
CM + CCl4	301 ± 8.5^b^	5.9 ± 0.2^b^	2.6 ± 0.10^a^	56.0 ± 3.0^b^	46.7 ± 1.2^b^

Each value represents the mean ± standard error of means (SEM) of 6 rats.

^a^ Significantly different from control group(*p* < 0.05).

^b^ Significantly different from CCl_4_ group (*p* < 0.05).

*C*: Control, *CM*: camel milk, *CCl*_*4*_: Carbon tetrachloride.

**Table 4:** **Effects of CCl**_**4**_**and camel milk on minerals profile in rat**

Groups	Parameters			
	Calcium mg/dl	Phosphorus mg/dl	Magnesium mg/dl	Chloride mEq/L
C	9.5 ± 0.5	1.9 ± 0.5	4.4 ± 0.2	57.5 ± 0.5
CM	9.9 ± 0.1	2.5 ± 0.1	4.5 ± 0.1	57.0 ± 1.0
CCl4	10.0 ± 0.1	2.5 ± 0.1	4.2 ± 0.2	56.0 ± 1.7
CM + CCl4	10.3 ± 0.1	2.5 ± 0.1	4.2 ± 0.2	58.3 ± 1.0

Each value represents the mean ± standard error of means (SEM) of 6 rats.

*C*: Control, *CM*: camel milk, *CCl*_*4*_: Carbon tetrachloride.

### Histopathological findings

**Figure 1:**
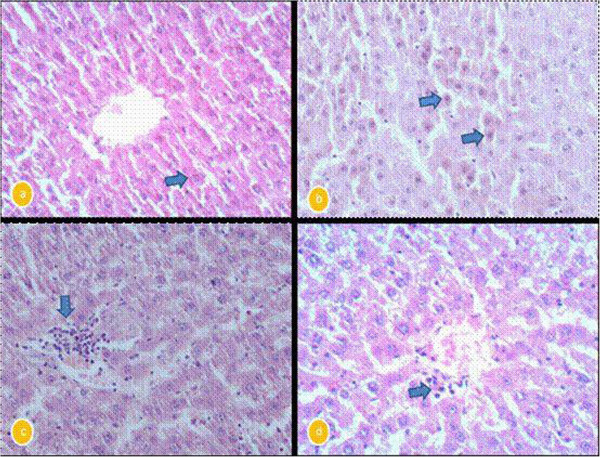
**a: liver of Camel milk only treated rats showing normal central veins and normal hepatocytes (arrow).** H&E X400. **b**: liver of carbon tetra chloride-intoxicated rats showing massive number of apoptotic hepatocytes (arrows). H&E X400. **c**: liver of carbon tetra chloride-intoxicated rats showing mononuclear cells infiltration in portal area (arrow). H&E X400. **d**: liver of camel milk + CCl4 treated rats showing few number of mononuclear cells around central veins. H&E X400.

The liver of control rats revealed that hepatocytes, portal triads and vasculature appeared within normal limit. The liver of camel milk only treated rats did not reveal any pathological alterations (necrosis, inflammation or fibrosis) (Figure 1a). The liver of CCl_4_-intoxicated rats showed massive fatty change and centrilobular necrosis in most cases. Additionally, single hepatic cell necrosis (apoptosis) was clearly observed in some cases (Figure 1b). Hepatitis characterized by mononuclear cells infiltration mostly macrophages and lymphocytes around central veins and in portal areas was also noticed in most cases of CCl_4_ intoxicated rats (Figure 1c).

The liver of CCl_4_-intoxicated rats and treated with camel milk exhibited clear hepatic recovery characterized by a complete regeneration of hepatocytes and the hepatic tissue appeared more or less normal in most cases (Figure 1d).

## Discussion

In the present study serum hepatic biomarkers, AST and ALT activities were greatly increased (p < 0.05) in rats with the CCl_4_ treatment rats compare to control. The increased serum levels of hepatic markers have been attributed to the liver injury, because these enzymes are place in cytoplasmic area of the cell and are released into circulation in case of cellular damage (Recknagel et al. 1989; Brent and Rumack 1993).

Zimmerman et al. (1965) stated that the CCl_4_ induced the increase of serum ALT and AST levels which source from cell membrane and mitochondrial damages in liver cells. There are many authors’ reports indicating that these enzymes activities were significantly elevated after CCl_4_ treatment (Tribble et al. 1987; Wang et al. 1997; Mehmetcik 2008; Arici and Cetin 2011). The first reports about hepatotoxic effects by CCl_4_ are lipid peroxidation origin, and are largely due to its active metabolite CCl_3_ (This metabolite can abstract hydrogen from fatty acids, initiating the lipid peroxidation), lead to cell injury, and finally liver damage (Forni et al. 1983; Park et al. 2005). On the other hand, treatment with camel milk was found to suppress (p < 0.05) the increase of serum AST and ALT activities induced by CCl_4_ treatment in rats. This finding implies that camel milk challenge to protect liver tissue from CCl_4_ injury. The reversal of increased serum enzymes in CCl_4_-induced liver damage by camel milk may be due to the prevention of the leakage of intracellular enzymes by its membrane stabilizing activity. This is in agreement with the commonly accepted view that serum levels of transaminases return to normal with the healing of hepatic parenchyma and the regeneration of hepatocytes (Thabrew and Joice 1987). Several studies have provided a considerable support for evidencing the protective effects of camel milk on liver damage (Hamad et al. 2011; Khan and Alzohairy 2011; Al-Fartosi et al. 2012). Also, these studies declared that the protective effect of camel milk against CCl_4_-induced oxidative stress in the rat is due to its antioxidant properties. Camel milk was found to contain high concentrations of vitamins A, B_2_, C and E and is very rich in magnesium and other trace elements, these vitamins act as antioxidants and have been found to be useful in preventing toxicant-induced tissue injury (Yousef 2004). The efficacy of any hepatoprotective drug is dependent on its capacity of either reducing the harmful effect or restoring the normal hepatic physiology that has been distributed by a hepatotoxin. Camel milk decreased (p < 0.05) CCl_4_ induced elevated enzyme levels in tested groups, indicating the protection of structural integrity of hepatocytic cell membrane or regeneration of damaged liver cells (Palanivel et al. 2008).

As in our experiments, previous experimental studies have shown that CC1_4_ increased significantly serum ALP (Khan and Al-Zohairy 2011) levels, and decreased urea (Sreepriya et al. 2001; Khan and Al-Zohairy 2011), total protein (Fahim et al. 1999; Khan and Al-Zohairy 2011) and albumin (Fahim et al. 1999; Khan and Al-Zohairy 2011) levels. However, there is a controversy about the effect of CC1_4_ on serum creatinine level. While some investigators (Cruz et al. 1993) found a decrease in serum creatinine in CCl_4_ toxicity, parallel to the present study others (Wirth et al. 1997; Palaparthy et al. 2001; Ozdogan et al. 2002; Khan and Al-Zohairy 2011) found no significant changes. In addition current study, reported that calcium, phosphorus, chloride and magnesium values in CC1_4_ administrated rats were not statistically different (p > 0.05) from control values (Ogeturk et al. 2004).

In this study there was a significant (p < 0.05) decrease in serum albumin of rats treated with CCl_4_ (group 3) as compared to the control rats either received tape water (group 1) or camel milk (group 2). Indicating poor liver functions or impaired synthesis, either primary as in liver cells damage or secondary to diminished protein intake and reduced absorption of amino acids caused by a malabsorption syndromes or malnutrition, or loss protein in urine, due to nephritic syndrome and chronic glomerulonephritis (Al-Fartosi et al. 2012). On the other hand, a significant (p < 0.05) increase in concentration of serum albumin was observed in rats received camel milk either alone (group 2) or with CCl_4_ (group 4) compare to group 3 in which rats received CCl_4_ only. The increase of albumin concentration after treatment with camel milk may be attributed to the decrease in lipid peroxidation processes and increase in the activities of plasma protein thiols as a result of treatment with camel milk in both animal and human (Al-Hashem et al. 2009; Al-Fartosi et al. 2012).

In the present study, serum glucose value was reduced (p < 0.05) in CCl_4_-treated rats. This decrease was restored towards the control value when CCl_4_ intoxicated rats treated with camel milk. Studies have demonstrated decreased hepatic glycogen content after treatment with CCl_4_, reflecting decreased gluconeogenesis by the liver (Muriel et al. 2001). It has been known that, hypoglycemia is main feature of CCl_4_ toxicity (Mion et al. 1996). The same author reported that, hypoglycemia was observed in liver cirrhotic rats. The potential hypoglycemic effect of camel milk observed in the current work was not out of expectation with respect to the highest insulin content obtained for camel milk as revealed from the data in Table 3. These findings is consistent with the observation of Agrawal et al. (2002), Agrawal et al. (2007) and Hamad et al. (2011) for hypoglycemic effect of camel milk. It should be noted that, camel milk does not form coagulum in acidic environment of stomach, which may in turn provides a rapid pass of camel milk with its specific like protein/insulin through stomach and remains available for absorption in intestine (Hamad et al. 2011).

The results of the present study have also established that, the CCl_4_ treatment could have affected the lipid metabolism of liver (triglyceride and cholesterol levels). This is evidenced from the present observations that, CCl_4_ caused a significant (p < 0.05) increase in the levels of lipid parameters. Muller et al. (1974) stated that CCl_4_ intoxication is similar to hepatitis in case of the triglycerides catabolism. This situation could be also attributed to the reduction of lipase activity, which could lead to decrease in triglyceride hydrolysis (Jahn et al. 1985). On the other hand, it can be assumed that hypercholesterolemia in CCl_4_ intoxicated rats was resulted from damage of hepatic parenchymal cells that lead to disturbance of lipid metabolism in liver (Havel et al. 1986). However, rats treated with camel milk showed a significant (p < 0.05) decline in triacylglycerol and cholesterol values compared to CCl_4_-intoxicated rats. The mechanism of lipid lowering effects of camel milk might be attributed to an inhibitory activity on microsomal acyl coenzyme A: cholesterol acyltransferease in vitro. This enzyme is responsible for acylation of cholesterol to cholesterol esters in liver (Matsuda 1994).

The biochemical findings were also confirmed by histological observations. The changes mostly include hepatocellular necrosis or apoptosis, fatty accumulation, inflammatory cells infiltration and other histological manifestations which were also consistent with the findings of other authors (Brattin et al. 1985; Sun et al. 2001; Khan and Al-Zohairy 2011).

## Conclusion

In conclusion, camel milk caused a protective effect against CCl_4_-induced liver damage and improved the biochemical parameters. Also, camel milk has a hepatoprotective effect against injury in the liver of CCl_4_-treated rats. Therefore, camel milk may be used to protect against toxic effects of CCl_4_ and other chemical agents in liver. In the future, examination of the liver protective effect of camel milk against CCl_4_ in dose dependant manner could be investigated.
